# The effect of current *Schistosoma mansoni* infection on the immunogenicity of a candidate TB vaccine, MVA85A, in BCG-vaccinated adolescents: An open-label trial

**DOI:** 10.1371/journal.pntd.0005440

**Published:** 2017-05-04

**Authors:** Anne Wajja, Dennison Kizito, Beatrice Nassanga, Angela Nalwoga, Joyce Kabagenyi, Simon Kimuda, Ronald Galiwango, Gertrude Mutonyi, Samantha Vermaak, Iman Satti, Jaco Verweij, Edridah Tukahebwa, Stephen Cose, Jonathan Levin, Pontiano Kaleebu, Alison M. Elliott, Helen McShane

**Affiliations:** 1 Co-infection Studies Program, MRC/UVRI Uganda Research Unit, Entebbe, Uganda; 2 The Jenner Institute, University of Oxford, Oxford, United Kingdom; 3 Laboratory for Medical Microbiology and Immunology & Laboratory for Clinical Pathology, St. Elisabeth Hospital, Tilburg, The Netherlands; 4 Vector Control Division, Ministry of Health, Kampala, Uganda; 5 London School of Hygiene & Tropical Medicine, London, United Kingdom; 6 School of Public Health, Faculty of Health Sciences, University of the Witwatersrand, Johannesburg, South Africa; George Washington University School of Medicine and Health Sciences, UNITED STATES

## Abstract

**Introduction:**

Helminth infection may affect vaccine immunogenicity and efficacy. Adolescents, a target population for tuberculosis booster vaccines, often have a high helminth burden. We investigated effects of *Schistosoma mansoni* (Sm) on the immunogenicity and safety of MVA85A, a model candidate tuberculosis vaccine, in BCG-vaccinated Ugandan adolescents.

**Methods:**

In this phase II open label trial we enrolled 36 healthy, previously BCG-vaccinated adolescents, 18 with no helminth infection detected, 18 with Sm only. The primary outcome was immunogenicity measured by Ag85A-specific interferon gamma ELISpot assay. Tuberculosis and schistosome-specific responses were also assessed by whole-blood stimulation and multiplex cytokine assay, and by antibody ELISAs.

**Results:**

Ag85A-specific cellular responses increased significantly following immunisation but with no differences between the two groups. Sm infection was associated with higher pre-immunisation Ag85A-specific IgG4 but with no change in antibody levels following immunisation. There were no serious adverse events. Most reactogenicity events were of mild or moderate severity and resolved quickly.

**Conclusions:**

The significant Ag85A-specific T cell responses and lack of difference between Sm-infected and uninfected participants is encouraging for tuberculosis vaccine development. The implications of pre-existing Ag85A-specific IgG4 antibodies for protective immunity against tuberculosis among those infected with Sm are not known. MVA85A was safe in this population.

**Trial registration:**

ClinicalTrials.gov NCT02178748

## Author summary

There is an urgent global need for an improved TB vaccine strategy. Adolescents are an important target population for a TB vaccine. In field settings where the need for a vaccine is greatest, co-infection with other pathogens including malaria, HIV and helminths, may interfere with the impact of such strategies. In this study, we used a model TB vaccine candidate based on a genetically engineered viral vector, and with an excellent safety profile, to determine whether there was any immunological interference when this vaccine was used in adolescents who were co-infected with *S*. *mansoni*. Our data shows comparable immunogenicity in adolescents infected and uninfected with *S*. *mansoni*, suggesting that co-infection with this helminth species may not have an adverse impact on candidate TB vaccines of this type. The vaccine was safe and induced robust cellular responses in both the infected and uninfected adolescents following immunisation. These findings are important and encouraging for tuberculosis vaccine development.

## Introduction

Helminth infection is widespread with over a billion people infected worldwide [1]. In 2014, at least 258 million people required treatment for schistosomiasis, 90% of whom lived in Africa [2]. In animal models, schistosomiasis impairs responses to immunisation against hepatitis B [3], malaria [4], mycobacterial infection and BCG immunisation [5, 6]. In humans, the effects are less clear. Helminth infections can modulate vaccine-induced responses against tetanus [7], influenza [8], cholera [9], and *Salmonella typhi* [10]. T-helper (Th)1 responses are important for protective immunity against intracellular bacteria and viruses, while helminths characteristically induce Th2 [11] and immunoregulatory responses, both of which can down-regulate Th1 pathways [8]. If concurrent helminth infection impairs vaccine-induced immunity, policies for helminth treatment before, or at, the time of immunisation may be needed.

Tuberculosis (TB) remains a global health problem with very high mortality and morbidity. In 2014, there were an estimated 9.6 million new cases and 1.5 million deaths from the disease [12]. The only licensed TB vaccine, Bacille Calmette-Guérin (BCG), prevents severe disease in childhood [13–15] but does not protect against pulmonary TB in many parts of the world. Development of an effective vaccine is a key strategy for combating the epidemic.

Adolescents are an important target population for a TB vaccine [16, 17] because TB presents as the pulmonary (transmissible) form, in adolescents and young adults. However, adolescents harbour a high prevalence and intensity of helminth infection in countries where helminths are endemic.

MVA85A, a recombinant Modified Vaccinia Ankara virus expressing the *Mycobacterium tuberculosis* (*Mtb)* antigen 85A, is a clinically advanced TB vaccine candidate. It has been administered to over 2400 human subjects and has an excellent safety profile. It is highly immunogenic in BCG-primed UK adults [18], but only modestly immunogenic in BCG-vaccinated South African infants [19], and did not confer protection in either BCG-vaccinated infants or HIV-infected adults [20]. Reasons for the reduced immunogenicity, and methods of enhancing immunogenicity, are currently being evaluated [21]. Co-infection with helminths may contribute to poor immunogenicity in high TB burden countries.

We here use MVA85A, a candidate TB vaccine with an excellent safety profile, as a model TB vaccine candidate with which to evaluate the effect of *S*. *mansoni* (Sm) infection on the T cell immunogenicity induced. Findings from this study may be important for all TB vaccine development.

## Materials and methods

### Ethics statement

The study was approved by the Research Ethics Committee of the Uganda Virus Research Institute, the Uganda National Council for Science and Technology, the Uganda National Drug Authority, Oxford Tropical Research Ethics Committee, and London School of Hygiene & Tropical Medicine Ethics Committee. Participants provided written informed assent and their parents or guardians gave written informed consent.

### Study design

This was a phase II open-label, non-randomised trial with two trial groups: Group 1 without any current helminth infection detected and Group 2 with current Sm infection only. Laboratory staff performing immunological assays were blinded to helminth infection status.

The primary objective was to compare the T cell immunogenicity of MVA85A in adolescents with and without Sm infection. Secondary objectives were to explore effects of Sm infection on other aspects of the immune response following MVA85A immunisation and to assess the safety of MVA85A in BCG-vaccinated Ugandan adolescents.

### Study setting

The study took place in five primary schools within schistosomiasis-endemic areas in Wakiso District, Uganda, on the shores of Lake Victoria, between June 2014 and January 2015. Community leaders, district Ministry of Education and Health officials and school staff were consulted and parent-teacher meetings held before the trial started to describe the trial, explain procedures and answer questions.

### Pre-screening

Pre-screening, by Kato Katz microscopy, was undertaken by the Vector Control Division (VCD) of the Ministry of Health who hold the mandate to screen for helminths in Ugandan schools. Children who had no helminths (candidates for Group 1) or Sm only (candidates for Group 2), who were within the eligible age group and who had a BCG scar, were invited for screening visits.

### Recruitment and follow up

Assenting adolescents, with consent from a parent or guardian, were screened on the school premises. A maximum of 60 days was allowed between screening and vaccination. Participants provided blood for haematology and biochemistry, urine and three stool samples at screening, and blood samples for evaluation of immune responses at screening, enrolment and 7, 28 and 56 days post-vaccination. Stool samples for Kato Katz for all participants were performed at D28 and D56. Group 2 participants had an extra visit at D42 for anthelminthic treatment.

### Exclusion and inclusion criteria

Participants were eligible for vaccination if aged 12 to 17 years, resident in the study area, BCG-vaccinated (based on BCG scar or written documentation), healthy by history and physical examination. Exclusion criteria were clinical, radiological, or laboratory evidence of, or previous treatment for, active or latent TB (including a positive ELISpot for ESAT6 or CFP10 at screening); sharing a residence with an individual on anti-TB treatment or with culture or smear-positive pulmonary TB within the last year; positive serology for HIV (Murex Diasorin, Italy and Vironostika, Biomerieux, France), hepatitis B or C (Innotest HCV Ab IV, Innogenetics, Belgium); positive rapid diagnostic test for malaria (SD-Bioline Inc, Korea); *Mansonella perstans* infection (modified Knott’s method [22]); intestinal parasites other than Sm; pregnancy; history of anaphylaxis or allergy likely to be exacerbated by vaccine; haematological or biochemical findings deemed clinically significant at screening; Sm infection intensity>2000 eggs per gram of stool (these individuals were treated immediately).

### Definition of study groups

Three baseline stool samples were examined by the Kato Katz method [23, 24]. Urine was examined for schistosome circulating cathodic antigen (CCA) by rapid test (ICT International, Cape Town, South Africa). Participants were recruited to Group 1 (no helminths) if none of these assays were positive. Participants were recruited to Group 2 (Sm infected) if at least one stool sample was microscopy positive on Kato Katz examination for Sm, and none of the investigations were positive for any other helminths.

One stool sample per participant was also examined by PCR [25–28] for Sm, *Strongyloides stercoralis* and *Necator americanus* (helminths prevalent in the area [29, 30]). Participants were excluded from Group 1 if Sm positive and from both groups if *S*. *strongyloides* positive. The protocol initially required exclusion from both groups if PCR positive for *N*. *amercianus*, however, this was amended when a high proportion of Sm positive individuals were found to be *N*. *americanus* PCR positive (despite having three samples negative by microscopy).

### Investigational medicinal product

MVA85A was produced under Good Manufacturing Practice conditions by IDT Biologika GmbH, Dessau-Rosslau, Germany. It was stored at -80°C in a locked, temperature‐monitored freezer at the Uganda Virus Research Institute-International AIDS Vaccine Initiative HIV Vaccine Program pharmacy, Entebbe. On vaccination days, vaccine was transported from pharmacy to field on dry ice.

### Vaccination

All participants received 1x10^8^ plaque forming units (PFU) MVA85A as a single intramuscular injection in the deltoid region.

### Anti-helminthic treatment

All participants were treated for helminths with praziquantel (40mg/kg) and albendazole (400 mg) under observation by trial staff. Group 1 received a single dose on D56 (in accord with annual mass treatment performed by VCD). Group 2 participants received two doses of praziquantel, one on D28 (after samples were obtained) and one on D42, to optimise clearance of infection. In this community, free diagnosis and treatment for Schistosomiasis is largely provided by the VCD, with whom we worked, so any treatment was done with the study team’s knowledge.

### Endpoints

The primary endpoint was T cell immunogenicity assessed by *ex vivo* Ag85A-specific interferon gamma (IFN-γ) ELISpot response. Secondary endpoints were the profile of cytokine responses assessed by multiplex Luminex assay of supernatant following six-day whole blood stimulation, plasma antibody concentrations, and solicited and unsolicited local and systemic adverse events.

### Immunological assays

#### *Ex vivo* IFN-γ ELISpot assay

Antigen-specific IFN-γ secreting cells were detected by ELISpot on fresh peripheral blood mononuclear cells (PBMC), using IFN-γ ELISpot kits (Mabtech, Sweden) as previously described [18, 21, 31, 32]. Briefly, IFN-γ was assessed in PBMC (0.3x10^6^/well) in response to PPD-T (20μg/ml), ESAT-6 (10μg/ml), CFP-10 (10μg/ml) and Ag85A (a single 66 peptide pool; 2μg/ml). Media only and SEB (10μg/ml) were used as negative and positive controls. Plates were read using an ELISpot AID reader. Results were obtained by subtracting mean background responses in unstimulated wells from mean responses in stimulated wells and reported as spot-forming cells (SFC) per million PBMC.

### Whole blood stimulation and multiplex Luminex assay

Whole blood assays were conducted as previously described [18, 31, 33]. Briefly, whole blood diluted to a final concentration of 1:4 in RPMI supplemented with 10% Fetal Calf Serum (R10) was stimulated with Ag85A (2μg/ml), PPD-T (10μg/ml), schistosome worm antigen (SWA;10μg/ml, provided by Professor M Doenhoff, University of Nottingham, UK) and phytohaemaggluttin (Sigma-Aldrich; 10μg/ml) or left unstimulated and incubated for 6 days in 5% carbon dioxide at 37°C. Cytokine responses (IFN-γ, interleukin (IL)10, tumour necrosis factor (TNF)α, granulocyte-monocyte stimulating factor (GMCSF), IL12P40, IL13, IL17A, IL1A, IL2, IL5, inducible protein (IP)10, macrophage inflammatory protein-1-alpha (MIP1ɑ), IL6, IL1270, IL4, monocyte chemoattractant protein-3 (MCP3) and macrophage-derived chemokine (MDC)) in culture supernatants were assessed using a MILLIPLEX MAP Kit, (EMD Millipore Corporation, Billerica, MA, USA). Detection antibody cocktail, streptavidin-phycoerythrin working concentrations, and the bead cocktail were diluted to allow three plates to be run from each kit.

### Antibody ELISA

ELISA assays were used to measure levels of total IgG, IgG1, IgG4 and IgE to Ag85A, SWA and SEA using an established protocol modified to include responses to mycobacterial antigens [34], detailed in the supplementary methods (S1 Methods).

### Parasite PCR assays

A portion of a stool sample from each participant was suspended in ethanol and stored at -80°C. Subsequently, DNA was extracted and samples were examined for Sm, *S*. *stercoralis* and *N*. *americanus* DNA, as previously described [25].

Aliquots of EDTA-anticoagulated whole blood were stored at -80°C. To investigate for low-intensity *Plasmodium falciparum* infection (the common malaria species in this setting [35] which could have been missed by the rapid diagnostic test) DNA was extracted from 500μl whole blood then assessed using Real Time PCR as previously described [36] and detailed in the supplementary methods (S1 Methods). A positive malaria PCR was not an exclusion criterion for eligibility.

### Adverse events

Solicited and unsolicited adverse events were collected using a diary card completed at home by the participant from D0 to D6 and were assessed by the clinician at every clinic visit.

### Statistical methods

The initial plan was to enrol 30 subjects in group 1 and 40–60 subjects in group 2. The sample size was amended due to challenges with enrolment, to 12–24 subjects in each group. With 12–18 participants in each group, the study had 56–74% power to detect a 40% reduction in geometric mean SFUs, and 82–94% power to detect a 50% reduction. The sample size calculation was based on previous ELISpot data from an adolescent trial [37] and assumed a mean response on the log-scale for SFUs of 6.40 with a standard deviation of 0.59 corresponding to a geometric mean of 600 SFUs on the back-transformed original scale. The initial plan was to match participants in the two groups by age and gender, however, this was not possible and these variables were therefore considered *a priori* as confounders.

The primary immunogenicity analysis was comparison of Ag85A ELISpot responses between Sm infected (Group 2) and uninfected (Group 1) adolescents at peak response (D7). Non-parametric analyses using the Mann Whitney U-test and multiple linear regression with bootstrapped confidence intervals adjusting for age, gender, school and hookworm PCR results were used. Area under the curve (AUC) analysis was done from D0 to D28.

Multiplex Luminex data were analysed using R (version 3.2.2). Unstimulated cytokine responses were subtracted from antigen-stimulated results and negative values were assigned 0. Baseline responses were compared between the groups and change following vaccination was evaluated. Cytokine responses were compared between Sm infected and Sm uninfected individuals by fitting regression models which also contained terms for appropriate confounders [38], age, gender and school. Mean (geometric mean) fold differences were calculated. Principal component analysis was conducted on the standardised log AUC cytokine data. Bonferroni corrections were used to account for multiple testing.

For antibody concentrations, comparisons were made between baseline levels and change following vaccination.

Solicited and unsolicited adverse events overall and for each group were summarised by frequency and severity.

## Results

**Fig 1:**
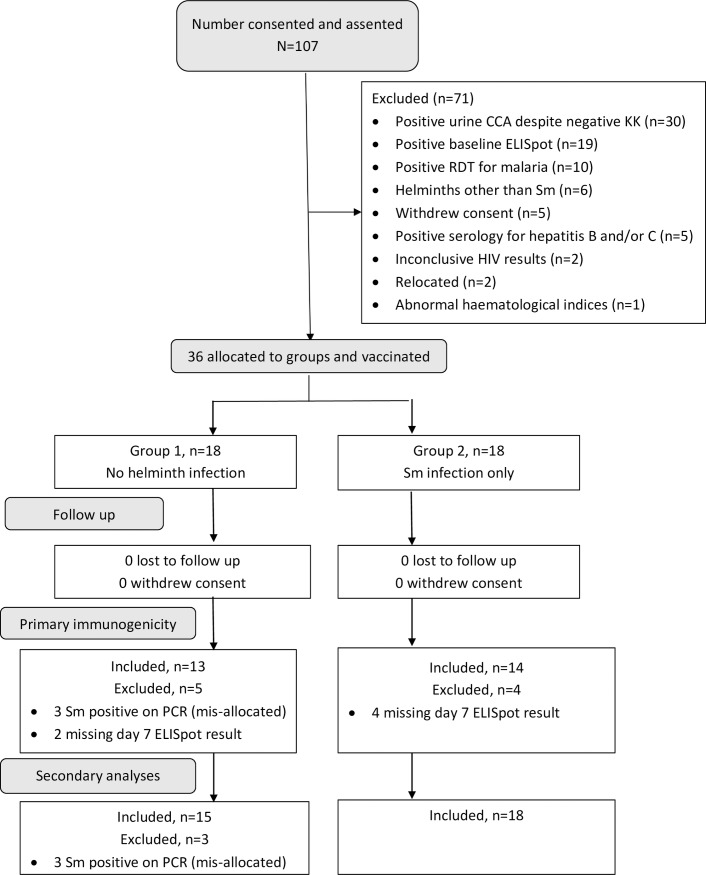
CONSORT flow diagram showing volunteer recruitment and follow up. Abbreviations: CCA, schistosome circulating cathodic antigen; KK, Kato Katz; RDT, rapid diagnostic test; Sm, *Schistosoma mansoni*.

1068 pupils were pre-screened, and 174, who were within the target age group of 12–17 years, were approached for screening. Sixty-seven were excluded before consenting, 35 because parents/guardians declined to consent; 12 had no BCG scar; 10 left school during the initial processes; five had no adult or responsible legal guardian accessible for consenting; three had recently been treated for schistosomiasis; one volunteered known HIV-positive status and one was found to be under age at screening (11 years) despite having reported being 12 years at pre-screening. 107 subjects consented, assented and were screened for eligibility of whom 36 were enrolled (Fig 1).

Eighteen subjects had Sm infection only and were enrolled into Group 2, 18 had no helminth infection detected and were enrolled into group 1 (3 of these subsequently had Sm detected on PCR and were excluded from all further analysis). Participants in Group 1 and 2 differed in age, school and *N*. *americanus* PCR status (Table 1).

**Table 1:** Baseline characteristics of eligible subjects by group.

Characteristics	Level	Group 1	Group 2
		No Helminths	Sm Only
		(n = 18)	(n = 18)
		n (%)	n (%)
**Age group (years)**	11–12	6 (33)	8 (44)
	13–14	11 (61)	7 (39)
	16–17	1(6)	3 (17)
**Gender**	Male	10 (56)	10 (56)
	Female	8 (44)	8 (44)
**School**	Busabala P/S	0 (0)	6 (33)
	Officers’ children P/S	1 (6)	1 (6)
	Kigo-Lunnya P/S	5 (28)	2 (11)
	Bulega P/S	6 (33)	9 (50)
	Bugiri P/S	6 (33)	0 (0)
**Mother’s education**	None	1 (6)	1 (6)
	Primary	10 (56)	13 (72)
	Secondary or higher	7 (39)	3 (17)
	Unknown	0 (0)	1 (6)
**Bathe in lake water**	No	12 (67)	4 (22)
	Yes	6 (33)	14 (78)
**Stool PCR for hookworm**	Positive	3 (17)	2 (11)
	Negative	15 (83)^a^	16 (89)
**Malaria PCR**	Positive	4 (22)^b^	1 (6)
	Negative	14 (78)^c^	17 (94)
**Intensity of Sm infection**	Uninfected	18 (100)	0 (0)
	Light (1–99 epg)	0 (0)	13 (72)
	Moderate (100–399 epg)	0 (0)	1 (6)
	Heavy (400–1999^d^ epg)	0 (0)	4 (22)
**Status for primary**	Excluded–due to helminths	3 (17)	0 (0)
**immunogenicity analysis of**	Day 7 ELISpot missing	2 (11)	4 (22)
**Ag85A**	Included	13 (72)	14 (78)

Abbreviations: *Sm*, *Schistosoma mansoni;* P/S, primary school; PCR, polymerase chain reaction; epg, eggs per gram of stool

a. Three excluded from immunogenicity analysis due to misallocation

b. Two excluded from immunogenicity analysis due to misallocation

c. One excluded from immunogenicity analysis due to misallocation

d. Individuals with egg counts ≥ 2000 epg were excluded and treated immediately.

### Effect of *S*. *mansoni* on vaccine immunogenicity

**Fig 2:**
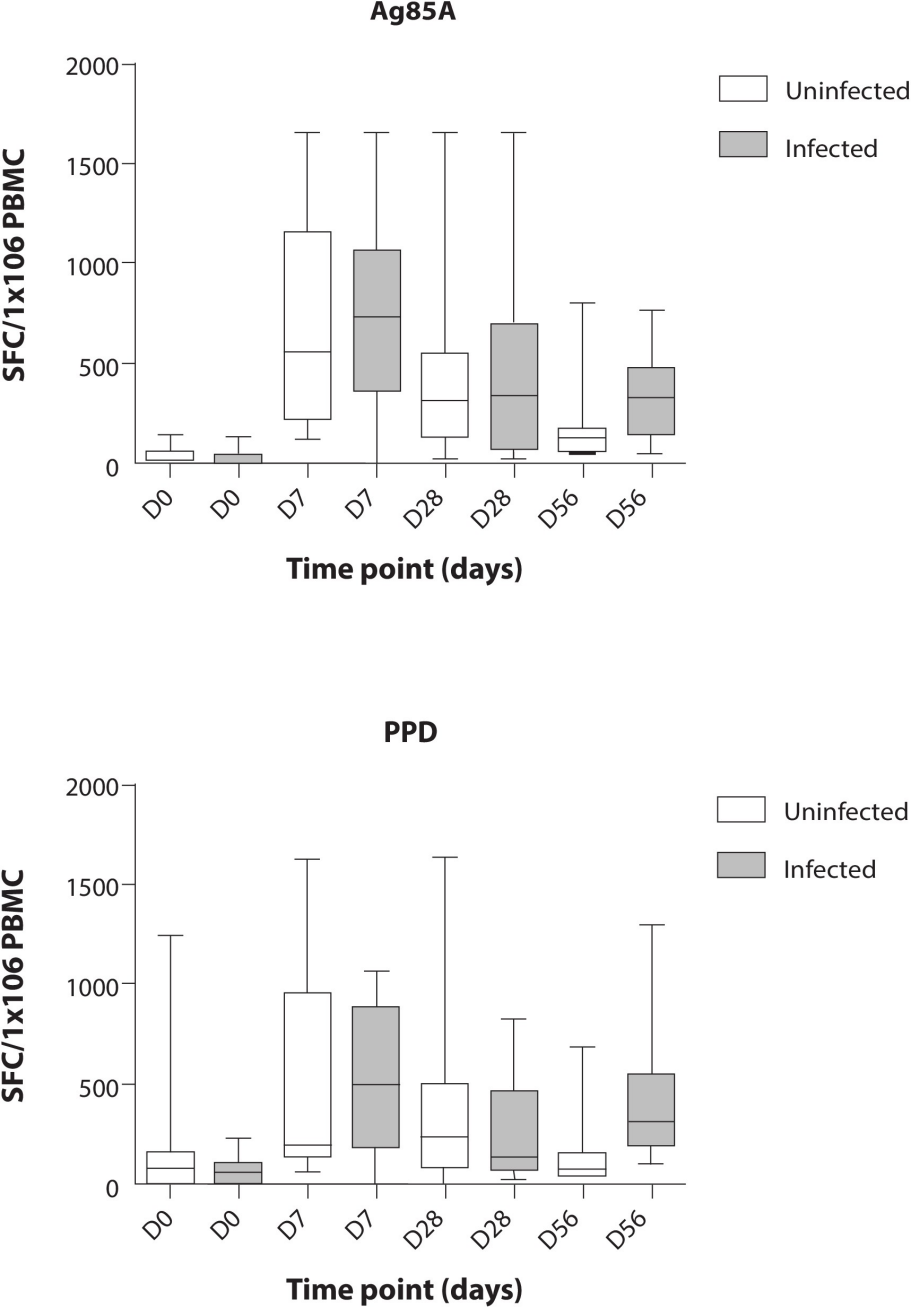
Ex-vivo interferon gamma responses to antigen 85A and purified protein derivative at each time point. Boxes in white represent uninfected children, grey represent Sm infected children. Boxes and whiskers show interquartile ranges and minimum and maximum values with horizontal lines representing medians. Participants in the Sm infected group were treated with praziquantel and albendazole twice between day 28 and day 56. Abbreviations: IFN-γ, interferon gamma; Ag85A, antigen 85A; PPD, purified protein derivative; Sm, *Schistosoma mansoni*.

The peak D7 Ag85A IFNγ ELISpot response was compared between groups. Two participants from group 1 and four from group 2 with missing data were excluded from the analysis. Median Ag85A SFC/ 1x10^6^ PBMC were not significantly different (Mann-Whitney p = 0.65). Regression and AUC analysis of the Ag85A ELISpot response (D0 to D28) adjusting for age, gender, school and stool PCR for *N*. *americanus* showed no evidence of an association between Sm infection and MVA85A induced IFNγ response (see Table 2 and Fig 2).

**Table 2:** Regression Analysis of Ag85A IFN-γ response as measured by ELISPOT on day 7 and Area Under the Curve analysis for days 0 to 28.

Factor	Level	Estimate (Bootstrap 95%	P-value
		C.I)	
**Ag85A at day 7**			
**Constant**		1272.3 (-2066.5; 4611.0)	-
**Age**	Per one year	-76.4 (-303.9; 151.2)	0.51
	increase		
**Sex**	Male	0 (Ref)	0.41
	Female	232.4 (-318.8; 783.1)	
**School**	Busabala	0 (Ref)	0.12
	Officers’ children	-225.6 (-1432.9; 981.8)	
	Kigo-Lunya	35.2 (-1055.7; 1126.1)	
	Bulega	471.9 (-346.0; 1289.7)	
	Bugiri	563.3 (-750.2; 1876.9)	
**Hookworm stool**	Negative	0 (Ref)	0.67
**PCR**	Positive	216.0 (-735.8; 1167.8)	
**Helminth Group**	No helminth	0 (Ref)	0.65
	*S*. *mansoni* only	185.1 (-607.3; 977.4)	
**AUC for Ag85A (day 0-day 28)**			
**Constant**		14 692 (-90 254; 119 639)	-
**Age**	Per one year	-821 (-8 565; 6 923)	0.84
	increase		
**Sex**	Male	0 (Ref)	0.68
	Female	3 018 (-11 301; 17 336)	
**School**	Busabala	0 (Ref)	0.32
	Officers’ children	43 (-27 250; 27 336)	
	Kigo-Lunya	4 839 (-19 193; 28 872)	
	Bulega	7 219 (-12 637; 27 075)	
	Bugiri	20 711 (-6 649; 48 071)	
**Hookworm stool**	Negative	0 (Ref)	0.92
**PCR**	Positive	-1 118 (-24 189; 21 954)	
**Helminth Group**	No helminth	0 (Ref)	0.56
	*S*. *mansoni* only	5 673 (-13 511; 24 856)	

Abbreviations: Ag85A, Antigen 85A; IFN-γ, interferon gamma; C.I., confidence interval; PCR, polymerase chain reaction; *Sm*, *Schistosoma mansoni*; Ref, reference; AUC, Area under the curve

D7 ELISpot and AUC analysis (D0—D28) for purified protein derivative (PPD) responses showed no significant difference between the two groups (S1 Table). ESAT and CFP 10 responses remained negative at all time points (S1 Fig).

**Fig 3:**
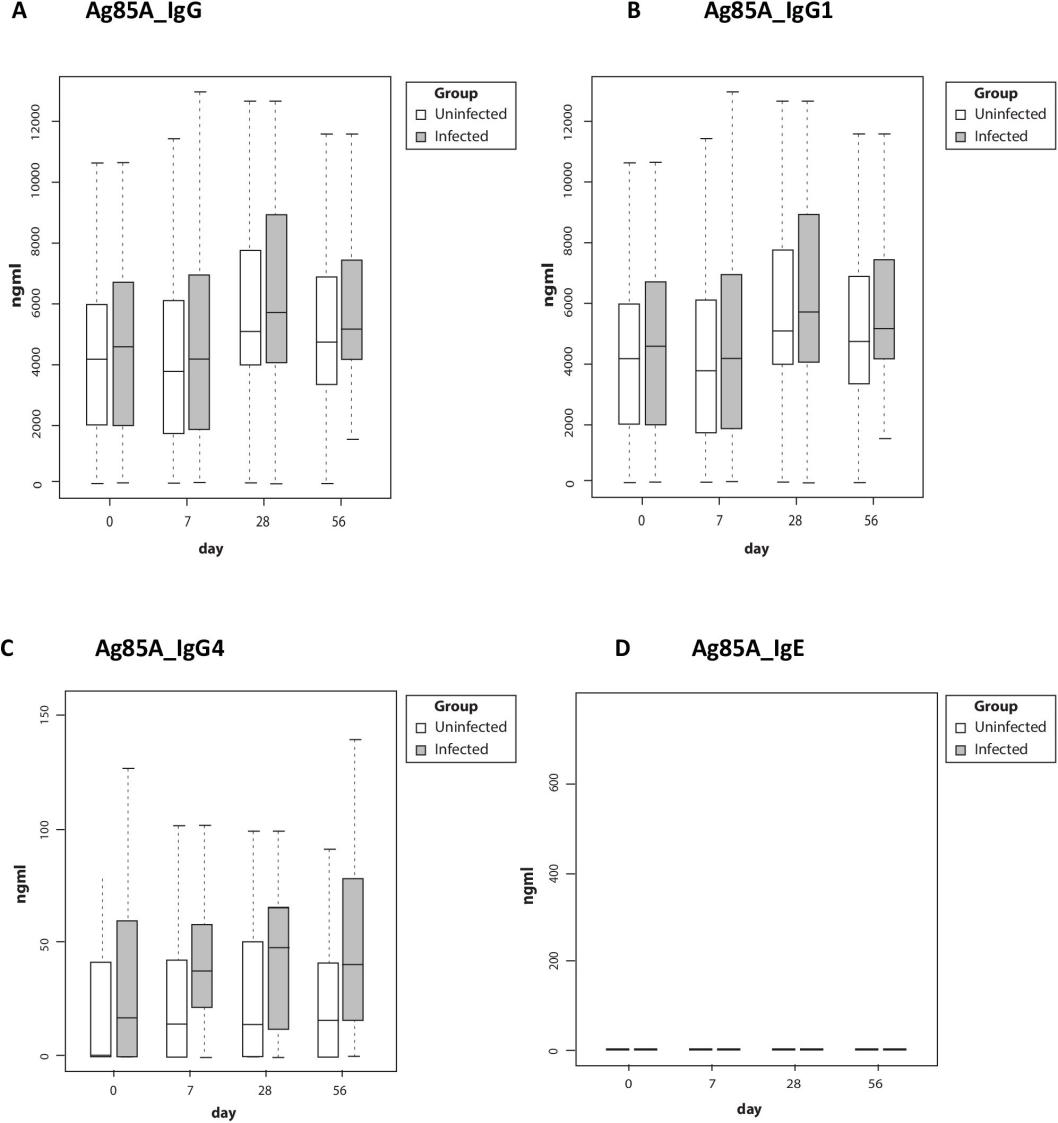
Antibody responses to *Mycobacterium tuberculosis* antigen 85A. Boxes in white represent uninfected children, grey represent Sm infected children. Boxes and whiskers show interquartile ranges and minimum and maximum values with horizontal lines representing medians. Participants in the Sm infected group were treated with praziquantel and albendazole twice between day 28 and day 56. Abbreviations: Ag85A, antigen 85A; Sm, *Schistosoma mansoni*

Ag85A antibody levels were not significantly different in the two groups with the exception of IgG4, which was significantly higher in the Sm infected group at baseline and at all time points post-vaccination (Fig 3); this difference remained significant after correcting for multiple testing using Bonferroni correction (p = 0.012). Ag85A-specific antibodies were present at baseline and showed no significant change post-vaccination. Our assay did not detect much Ag85A specific IgE in the study participants.

**Fig 4:**
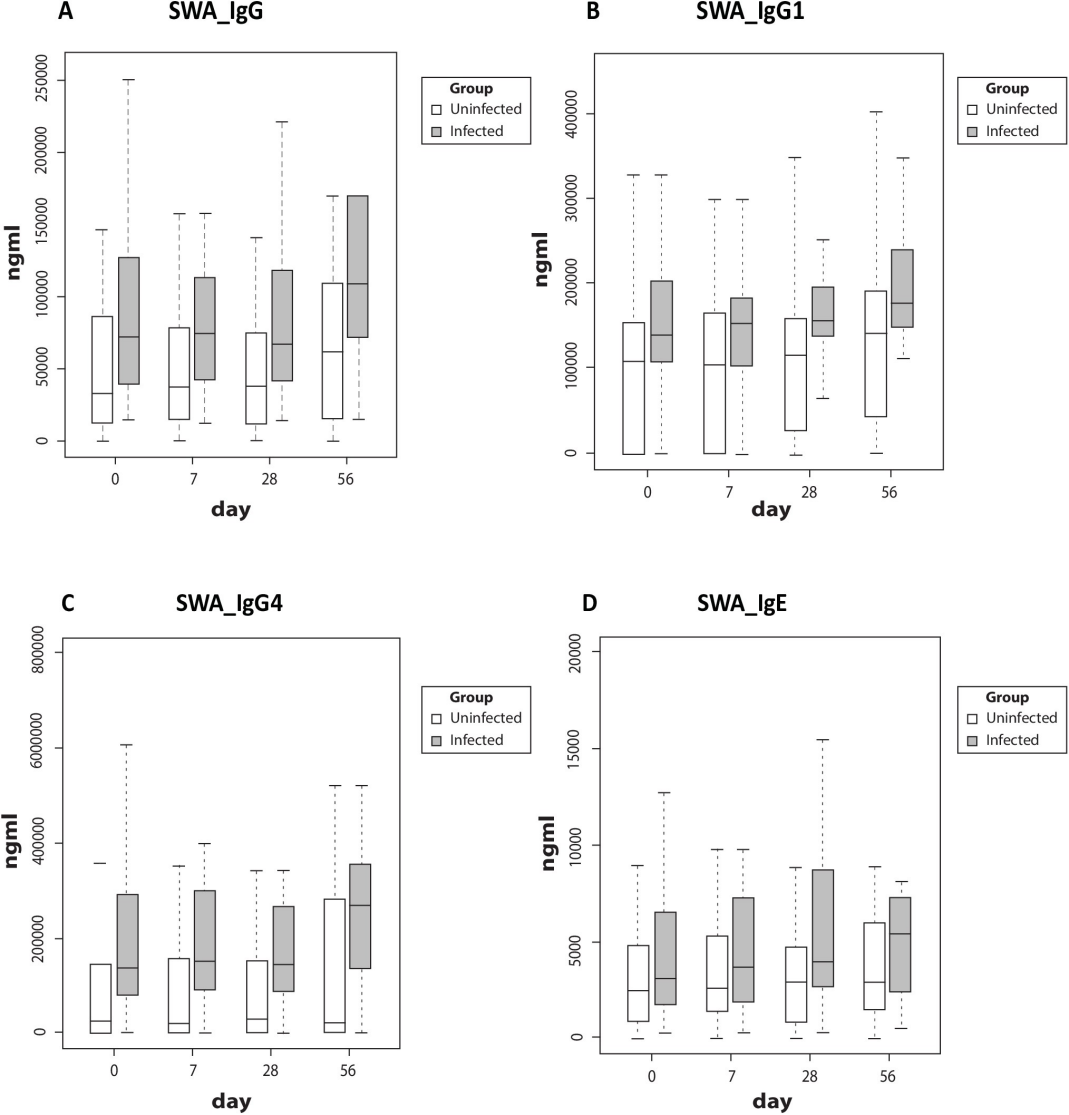
Antibody responses to schistosome worm antigen. Boxes in white represent uninfected children, grey represent Sm infected children. Boxes and whiskers show interquartile ranges and minimum and maximum values with horizontal lines representing medians. Participants in the Sm infected group were treated with praziquantel and albendazole twice between day 28 and day 56. P values for statistically significant differences in SWA-specific antibody responses between the groups were 0.0060 for IgG, 0.0136 for IgG1 and 0.0004 for IgG4. Abbreviations: SWA, schistosome worm antigen; Sm, *Schistosoma mansoni*

SWA-specific IgG antibodies and sub-classes were significantly higher (p values for IgG = 0.0060, IgG1 = 0.0136 and IgG4 = 0.0004) in the Sm infected group (Fig 4). SWA-specific IgE levels were low in both groups, and no statistically significant differences were seen between the groups (Fig 4). SWA-specific antibody levels tended to increase at day 56 in the Sm infected group (after praziquantel treatment). Exploring the observed elevated levels of Ag85A-specific IgG4, we found no correlation between SWA or SEA-specific IgG4 and Ag85A-specific IgG4 among Sm-infected participants.

Analysis of the multiplex Luminex assays following six-day stimulation and culture for Ag85A and PPD stimulation showed no significant difference in pre-immunisation responses for any cytokine, and all cytokines showed an enhanced response post-vaccination in both groups, with the exception of IL2, for which measured levels were very low. There was no significant difference post-vaccination in levels of any individual cytokine. Regression analysis (supplementary S3 and S4 Tables) of AUC accounting for age, gender and school as confounders suggested a higher response to Ag85A and PPD among Sm infected compared to uninfected participants for all cytokines except IL12P40 (to Ag85A) and IL2 (to both Ag85A and PPD). There was no significant difference in geometric means following stimulation with Ag85A and PPD for any of the cytokines (except for IL1A to PPD, p value = 0.017) between uninfected and infected when adjusted for multiple testing using Bonferroni correction (S3 and S4 Tables).

### Safety results

Safety results were considered for all 36 subjects who received the vaccine despite the fact that three subjects initially allocated to Group 1 were found to have Sm on PCR and therefore excluded from the immunogenicity analysis (Table 3 and S2 Table).

**Table 3:** Number and percentage of subjects reporting each adverse event

		Group 1 (No Helminths) (n = 18)^a^					Group 2 (Sm only)				
							(n = 18)				
**Adverse events**		Day	Diary	Day	Day	Day	Day	Diary	Day	Day	Day
		0	card	7	28	56	0	Card	7	28	56
**Solicited local**	Local pain	7(39)	15(83)	2(11)	0(0)	0(0)	3(17)	18(100)	1(6)	0(0)	0(0)
**AEs**											
	Local warmth	17(94)	11(61)	1(6)	0(0)	0(0)	17(94)	9(50)	0(0)	0(0)	0(0)
	Local swelling	1(6)	0(0)	2(11)	0(0)	0(0)	2(11)	0(0)	2(11)	0(0)	0(0)
	Local itching	2(11)	8(44)	0(0)	0(0)	0(0)	1(6)	7(39)	0(0)	0(0)	0(0)
	Scaling	0(0)	1(6)	3(17)	0(0)	0(0)	0(0)	2(11)	2(11)	1(6)	0(0)
**Unsolicited AEs**			11(61)					9(50)			
**Solicited**	Documented	2(11)	0(0)	0(0)	0(0)	0(0)	0(0)	0(0)	0(0)	0(0)	0(0)
**systemic AEs**	fever										
	Symptoms of	0(0)	11(61)	0(0)	0(0)	0(0)	0(0)	11(61)	0(0)	0(0)	0(0)
	feverishness										
	Malaise	0(0)	0(0)	0(0)	0(0)	0(0)	0(0)	0(0)	0(0)	0(0)	0(0)
	Arthralgia	0(0)	8(44)	2(11)	0(0)	0(0)	0(0)	6(33)	0(0)	0(0)	0(0)
	Headache	2(11)	13(72)	0(0)	2(11)	0(0)	0(0)	12(67)	0(0)	0(0)	0(0)
	Myalgia	0(0)	5(28)	2(11)	0(0)	0(0)	0(0)	5(28)	0(0)	0(0)	0(0)
	Fatigue	1(6)	10(56)	1(6)	0(0)	0(0)	0(0)	7(39)	1(6)	0(0)	0(0)
	Nausea	1(6)	5(28)	0(0)	0(0)	0(0)	0(0)	4(22)	0(0)	0(0)	0(0)

Abbreviations: Sm, *Schistosoma mansoni*; AEs, Adverse events

a. Includes three participants subsequently excluded from the immunogenicity analysis due to misallocation

No serious adverse events were reported in this trial. Using data collected from the diary cards and at clinic visits on days 0, 7, 28 and 56, all 36 children reported at least one adverse event. 34 (94%) of 36 children vaccinated experienced at least one local adverse event and 6 (17%) experienced at least one systemic event. There was no difference in adverse events between the two groups. All adverse events had resolved by day 56.

Local warmth (94%) and pain (92%) at the injection site were the most commonly reported of the solicited local adverse events by subjects in both groups, while headache (69%) was the most commonly reported of the solicited systemic adverse events in the diary card. The AE profile was not different in those excluded from the immunogenicity analysis.

259 graded adverse events were recorded (See S2 Table). 192 (72%) of the adverse events were mild. Two severe adverse events occurred and were pain at vaccination site and itching at vaccination site, both observed in participants in the no helminth group.

## Discussion

MVA85A induced a robust cellular response, with no difference in cellular immunogenicity between adolescents with and without current *Sm* infection. MVA85A did not boost the baseline Ag85-specific antibody response (as expected [18]). Levels of Ag85A specific IgG4 antibody were elevated among the Sm infected participants at baseline and at all subsequent time points. The safety profile was consistent with other published data on this vaccine from African populations [37, 39].

The peak (D7) Ag85A ELISpot response of >500 SFC/million PBMC, was comparable to, or higher than, responses previously observed among adolescents and adults in the Cape region of South Africa [37, 39], where lifetime exposure to schistosomiasis and other tropical parasitic infections is likely to be lower than in tropical Africa [40]. Current exposure to Sm among Ugandan adolescents did not impair the cellular immunogenicity of MVA85A. This is important given the poor efficacy of BCG in tropical latitudes [41]. One potential advantage for booster TB vaccines may be prior exposure to environmental mycobacteria: besides BCG, the Ugandan participants would have been extensively exposed to environmental mycobacteria [42], as evidenced by anti-mycobacterial antibody levels prior to MVA85A vaccination [18]. A point for caution is that selection for prior BCG based largely on BCG scar (as most had no records available) may have biased recruitment to individuals who received a more immunogenic formulation of BCG, or who were predisposed to stronger anti-mycobacterial responses, since scarring varies between BCG strains and only about 60% of infants scar in response to strains commonly used in Uganda [43].

Our initial focus was on Th1 cytokines as measured by ex-vivo ELISpot and Luminex, as there is clear evidence for a role for Th1 cytokines in protective immunity against *Mycobacterium tuberculosis*. Given the increased interest in a role of humoral immunity and B cells in protective immunity against mycobacteria, we also investigated the humoral response as measured by IgG to the Ag85A insert. Further measures of immunogenicity such as mycobacterial growth inhibition assays and other T cell functions may be considered as part of future work on stored samples from this trial. There is as yet no clearly defined immunological correlate of protection with which to measure candidate vaccine immunogenicity. However, the recent paper by Fletcher et al [44] indicated that pre-MVA85A-immunisation BCG-specific ELIspot responses and post MVA85A antibody (specific for antigen 85) were protective. We did not perform the former but did measure the latter and observed no effect.

Recruitment to this study was challenging and the final sample size had power only to detect a large effect of current Sm infection on vaccine immunogenicity. However, there is no suggestion in the data that Sm was associated with a reduced or biased response to immunisation. If anything, cellular responses to MVA85A were slightly stronger, and increased further following treatment with praziquantel, in the Sm infected group. This was surprising given prior results in animal models of schistosomiasis and BCG immunisation and infection [5, 6]. Our findings in the human population may relate, again, to the impact of prior mycobacterial exposure, to intensity of Sm infection (low in most of our subjects) and to the characteristics of MVA85A (a viral vector vaccine which enters host cells but does not replicate [18]) as opposed to BCG (an attenuated mycobacterial vaccine which replicates in the host).

Lack of bias in the profile of cytokine response following MVA85A was also surprising, given evidence from earlier studies of Th2 bias following tetanus immunisation among individuals with Sm infection [7], and more particularly given evidence of bias in the antibody response to Ag85A (to IgG4) prior to MVA85A administration.

The groups in this study were defined by detection of current Sm infection. It is possible that prior helminth exposure might influence vaccine responses (rendering current status less relevant). Recent helminthic disease acquisition in the Sm negative group during follow up, detectable by Kato Katz, was ruled out using the follow up samples collected. It is plausible that previous exposure to helminths in the Sm negative group (which we could not measure in this study) may be responsible for the lack of difference, but the differences in Sm-specific antibody levels make this unlikely. For the Sm positive group, we expect that infections were chronic–as we have previously reported among adults in related fishing communities [45]. The finding that schistosome-specific IgG levels were significantly higher in the Sm infected group suggests that these individuals differed in prior, as well as current, Sm exposure. Sm-specific IgE increases gradually among exposed populations. Low levels in these adolescents (Sm infected or otherwise) is in keeping with this pattern [46–49].

This was an observational study, susceptible to unmeasured confounding. We attempted to exclude confounding by parasitic co-infections by enrolling participants in whom no other parasites that are common in this setting could be detected. This proved challenging for sub-microscopic hookworm and malaria infection, but adjusting for hookworm PCR status and sub-microscopic malaria (measured by malaria PCR) made no difference to the results.

It may not be possible to extrapolate these findings to other vaccines and other species of helminth. There is substantial evidence of variability between populations in vaccine immunogenicity: notably, impaired responses to yellow fever vaccine were observed in Uganda, compared to Switzerland, in a study that found reduced vaccine virus replication and neutralising antibody production and persistence, in the context of elevated innate and adaptive immune response activation, among Ugandan volunteers [50]. There is evidence, for other vaccines, of differences (such as Th2 bias) in the response between people with and without schistosomiasis [7, 10], and of modulation of vaccine responses by other helminth species [8, 9]. Key factors in helminth-vaccine interactions are likely to include vaccine characteristics (live, replicating, protein or toxoid), prior exposure to environmental organisms or pathogens homologous to the vaccine, and the nature of the desired response (cellular or antibody).

We conclude that current Sm infection did not interfere with the cellular immunogenicity of MVA85A in our study population. These findings are important and encouraging for the development of TB vaccines in general, and support the further development of booster TB vaccines for populations in tropical countries. MVA85A was safe in this population of African adolescents.

## Supporting information

S1 Methods Supplementary methods. (DOCX)

S1 Fig Ex-vivo interferon gamma responses to ESAT-6 and CFP-10 at each time point. (DOCX)

S1 Table Regression Analysis of PPD response as measured by ELISPOT. (DOCX)

S2 Table Severity of graded adverse events. (DOCX)

S3 Table Regression analysis of AUC comparing geometric means of cytokine responses to Ag85A between the uninfected and infected groups as measured by multiplex Luminex assay. (DOCX)

S4 Table Regression analysis of AUC comparing geometric means of cytokine responses to PPD between the uninfected and infected groups as measured by multiplex Luminex assay. (DOCX)
