# Supplementary material for: The effect of current Schistosoma mansoni infection on the immunogenicity of a candidate TB vaccine, MVA85A, in BCG-vaccinated adolescents: An open-label trial
Source: PLoS Negl Trop Dis. 2017 May 4;11(5):e0005440. doi: 10.1371/journal.pntd.0005440 (PMC5417418; doi:10.1371/journal.pntd.0005440)
Supplement: S1 Methods — (DOCX) [file pntd.0005440.s001.docx]

**Supplementary methods**

**Antibody ELISA**

A protocol previously used by our group was modified to measure antibody responses to mycobacterial antigens [1]. Immulon® 4 HBX microtitre plates (Thermo Scientific, USA) were coated with 50µl per well of 10µg/ml of Ag85A (BEI Resources, National Institute of Allergy and Infectious Diseases, USA) or 8µg/ml of SWA or 2.4µg/ml of SEA and incubated at 4°C overnight. These plates were coated with serial dilutions of either human IgG (GenScript, USA), IgG1 (Sigma-Aldrich, USA), IgG4 (Sigma-Aldrich, USA) or IgE (Merck Millipore, USA) proteins of known concentration to allow conversion of optical density (OD) output into antibody concentration in ng/ml using standard curves. The plates were thereafter washed four times with phosphate buffered saline having 0.05 % Tween 20 (PBS-T), blocked with 150µl per well of 1% skimmed milk in PBS-T for 2 hours at room temperature and then washed thrice with PBS-T. Plasma samples were diluted in blocking buffer with dilutions of 1 in 400 for SWA IgG; 1 in 200 for Ag85 IgG and SWA IgG1; 1 in 40 for SWA and SEA IgG4 and IgE; 1 in 20 for Ag85A IgG1; 1 in 10 for Ag85A IgG4 and IgE assays. 50µl of diluted sample was added to Ag85A or SWA coated wells and the plates were incubated at 4°C overnight. The plates were washed four to six times with PBS-T and then 50µl per well of detection antibody diluted in blocking buffer was added. Anti-human IgG horseradish peroxidase (HRP; Dako, Denmark) was used at a concentration of 0.5g/ml as the detection antibody for the IgG assays. The plates were incubated with this antibody for one hour at room temperature. Biotinylated mouse anti-human IgG1, IgG4 and IgE (BD Pharmingen™, USA) were used at a concentration of 0.5µg/ml as the detection antibodies for the IgG1, IgG4 and IgE assays respectively. The plates were incubated with these antibodies at 4°C overnight and then washed six times with PBS-T before a further incubation with 50µl per well poly-HRP-streptavidin conjugate (Sanquin, The Netherlands) at a 1 in 4000 dilution for one hour at room temperature. The plates were then washed four to six times with PBS-T before detection of HRP enzyme activity with 100µl per well o-phenylenediamine (Sigma-Aldrich, USA) containing hydrogen peroxide. After a 15-minute incubation at room temperature the reaction was stopped by adding 25µl per well of 2 M sulphuric acid. The ODs of the different wells were read at 490nm test wavelength and a 630nm reference wavelength with an ELISA plate reader (BioTek Instruments, USA). The ODs were converted to ng/ml using standard curves and the result multiplied by assay specific sample dilution factors.

**DNA extraction and real time polymerase chain reaction (PCR) for parasites**

**Helminth PCR**

This was done as previously described [2]. Helminth DNA from one of the stool samples (suspended in ethanol and frozen at -80^o^C), was extracted using a QIAamp DNA Mini Kit (cat no 51306, Qiagen) with minor changes to manufacturer`s instruction as detailed below. Samples were left at room temperature to thaw before vortexing for 5 seconds, to homogenize the ethanol-faeces mixture. Half a millilitre of the homogenized mixture was transferred into an eppendorf tube and centrifuged at 13,000rpm for 3 minutes to get rid of the ethanol. The pellet was re-suspended in 200 μl of PBS with 2% polyvinylpolypyrolidone (777627, Fluka analytical, Sigma-Aldrich) and frozen overnight at -20 °C. Subsequently, the faeces-suspension was heated at 100°C for 10 minutes and a mixture of 200ul of ATL and proteinase K in a 9:1 ratio added. Samples were vortexed and incubated overnight at 55°C in heat block. 400 μl AL Buffer was then added and the sample incubated at 70°C for 10 minutes. This was followed by centrifugation for 1min at 13000rpm and transfer of the supernatant to 400 μl of ethanol (96-100 %).

The DNA was then purified in QIAamp spin columns using consecutive washes with 500ul AW1 and 500µl AW2 buffers. 200ul AE buffer was used to elute the DNA which was then quantified on a Nano drop 2000c. Specific forward and reverse primers and Taqman probes were used in a multiplex PCR to detect the different worm DNA simultaneously. Strongyloides stercoralis was detected using Stro18S-1530F, Stro18S-1630R and Stro18S-1586T with Quasar705 and (BHQ2); Necator americanus using Na58F, Na158R and Na81MGB with FAM and (BHQ1); Ancylostoma duodenale- Ad125F, Ad195R and Ad155-XS-YY with Yakima Yellow and (BHQ1) while Schistosoma mansoni was detected using Ssp48F, Ssp124R and Ssp78T-TR with Texas Red and (BHQ2). Phocin herpes virus DNA, extracted from the Phocin herpes virus (kindly provided by Dr. Martin Schutten, Erasmus Medical Center, Rotterdam, the Netherlands) was included in the PCR master mix thus distributed to all reaction wells as an internal control to check for PCR inhibition and proper interpretation of results. PhHV-267s, PhHVas and PhHV-305tq with Cy5 and (BHQ2) were used for Phocin herpes virus DNA detection. Serial dilutions of a positive pool were included on the plate for every run to allow setting a Ct value cut off for the test samples. The positive pool was made up of a mixture of DNA from samples (from one of our studies) that were highly positive for S. mansoni and N. americanus on Kato-Katz, and DNA positive for S. stercoralis and A. duodenale (kindly provided by Dr. Jaco J. Verweij, St. Elisabeth Hospital, Tilburg, the Netherlands). The amplification conditions were 10 minutes at 95^o^C, 50 cycles of 15 seconds at 95^o^C, 30s at 60^o^C and 30s at 72^o^C. DNA amplification, detection and data analysis were attained with the BIORAD CFX96 Real time system and Bio-Rad CFX manager Version 1.6.541.1028.

**Malaria PCR**

Malaria PCR was done for all the 36 volunteers as previously described [3]. First, DNA was extracted from 500ul whole blood (venous blood collected in EDTA anti-coagulant) using QIAamp 250 DNA Blood Mini Kit (QIAGEN, Germany) and eluted in a final volume of 100ul Buffer AE according to manufacturer’s instructions. The presence of P. falciparum in the extracted DNA was then assessed using Real Time PCR. Briefly, 2ul of sample DNA were added to 23ul Master Mix containing 10uM concentration of each Reverse (Plas 171R 5’-AAC CCA AAG ACT TTG ATT TCT CAT AA-3’) and Forward (PFal-F 5’CCG ACT AGG TGT TGG ATG AAA GTG TTA A-3’) primers for P. falciparum, 25uM concentration of each Reverse (PhHV-337as 5’-GCG GTT CCA AAC GTA CCA A-3’) and Forward (PhHV-267s 5’-GGG CGA ATC ACA GAT TGA ATC-3’) primers for Phocine Herpesvirus, 5uM P. falciparum probe and 25uM PhHV probe (Biolegio, Nijmegen, the Netherlands), 50mM MgCl2 (Sigma), HotStarTaq Master Mix (Qiagen) and Nuclease free water (Qiagen). White EU 8-tube strips SFGC and Natural EU 8-single attachable indented caps (Bioplastics BV, the Netherlands) were used. Each run consisted of 11 10-fold serial dilutions of DNA extracted from malaria positive (>1000mps/200WBCs confirmed on microscopy) sample, as positive controls and a negative control with nuclease free water in place of DNA. PhHV DNA was used as the internal control for the assay and it was added to the PCR Master Mix. All reactions were done in duplicate. C1000TM Thermocycler and CFX96 Real-time system software (BioRAD) were used for amplification, detection and analysis. The cycling conditions were: 15 min at 95^o^C followed by 50 cycles of 15s at 95^o^C, 30s at 60^o^C, and 30s at 72^o^C. Baseline threshold was set to begin at Ct 2 and end at 20. Samples were considered P. falciparum positive if mean Ct value was <40. Samples with just one amplification curve reaching the threshold line and/or with mean Ct value ≥40 were repeated at double volume and in triplicate. They were considered negative if mean Ct value remained ≥40. The assay was considered valid if efficiency was 100%±10%, R2 >0.98 and if PhHV internal control Ct values were consistent for all reactions.

**References**

1. Biraro IA, Egesa M, Kimuda S, et al. Effect of isoniazid preventive therapy on immune responses to mycobacterium tuberculosis: an open label randomised, controlled, exploratory study. BMC infectious diseases **2015**; 15:438.

2. Nampijja M, Webb EL, Kaweesa J, et al. The Lake Victoria Island Intervention Study on Worms and Allergy-related diseases (LaVIISWA): study protocol for a randomised controlled trial. Trials **2015**; 16:187.

3. Kaisar MM, Supali T, Wiria AE, et al. Epidemiology of Plasmodium infections in Flores Island, Indonesia using real-time PCR. Malaria journal **2013**; 12:169.
