# Supplementary material for: The effect of current Schistosoma mansoni infection on the immunogenicity of a candidate TB vaccine, MVA85A, in BCG-vaccinated adolescents: An open-label trial
Source: PLoS Negl Trop Dis. 2017 May 4;11(5):e0005440. doi: 10.1371/journal.pntd.0005440 (PMC5417418; doi:10.1371/journal.pntd.0005440)

**Supplementary Figure 1. Ex-vivo interferon gamma responses to ESAT-6 and CFP-10 at each time point.**

Boxes in white represent uninfected children, grey represent Sm infected children. Boxes and whiskers show interquartile ranges and minimum and maximum values with horizontal lines representing medians. Participants in the Sm infected group were treated with praziquantel and albendazole twice between day 28 and day 56.

Abbreviations: ESAT-6, early secretory antigenic 6 kDa; CFP-10, culture filtrate protein 10; Sm, *Schistosoma mansoni*


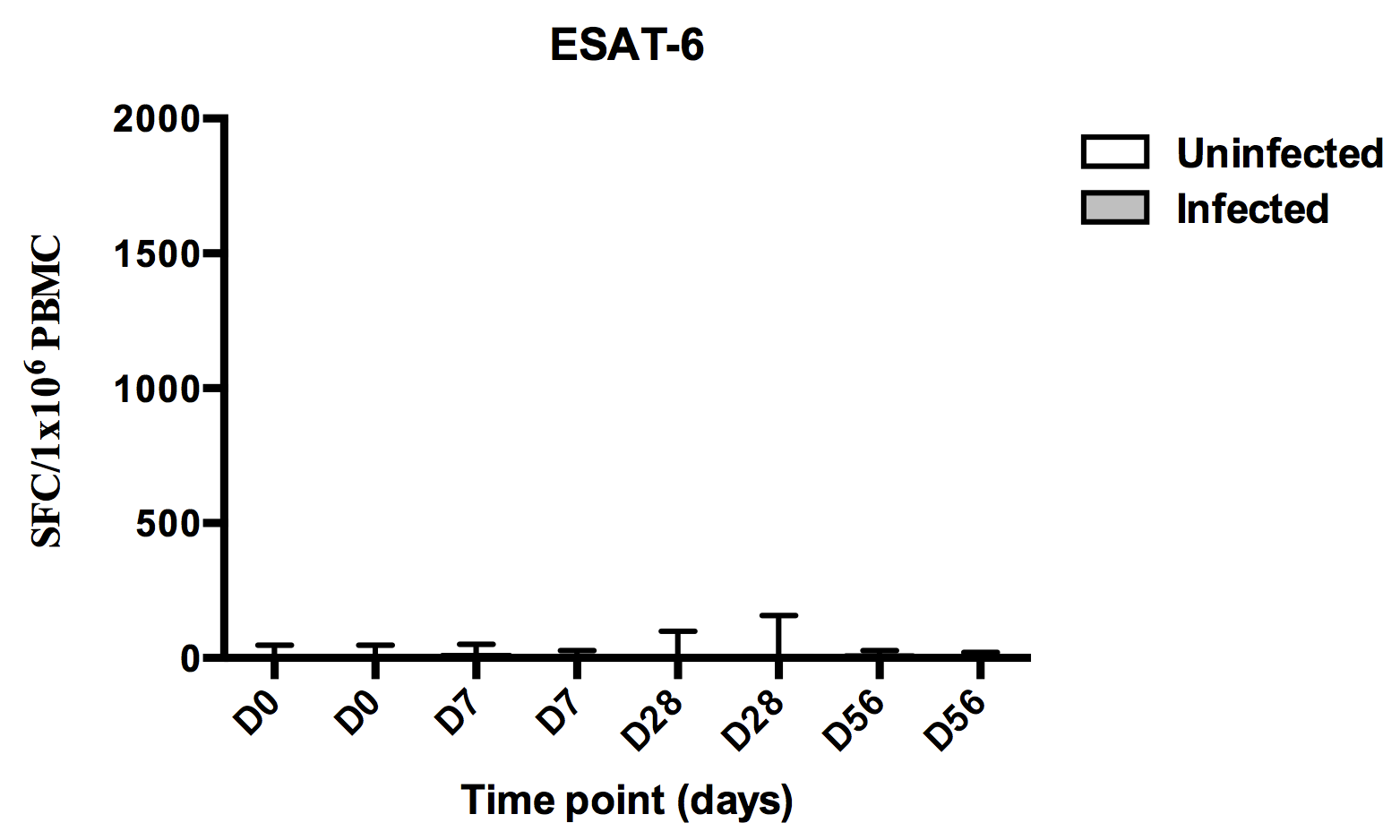


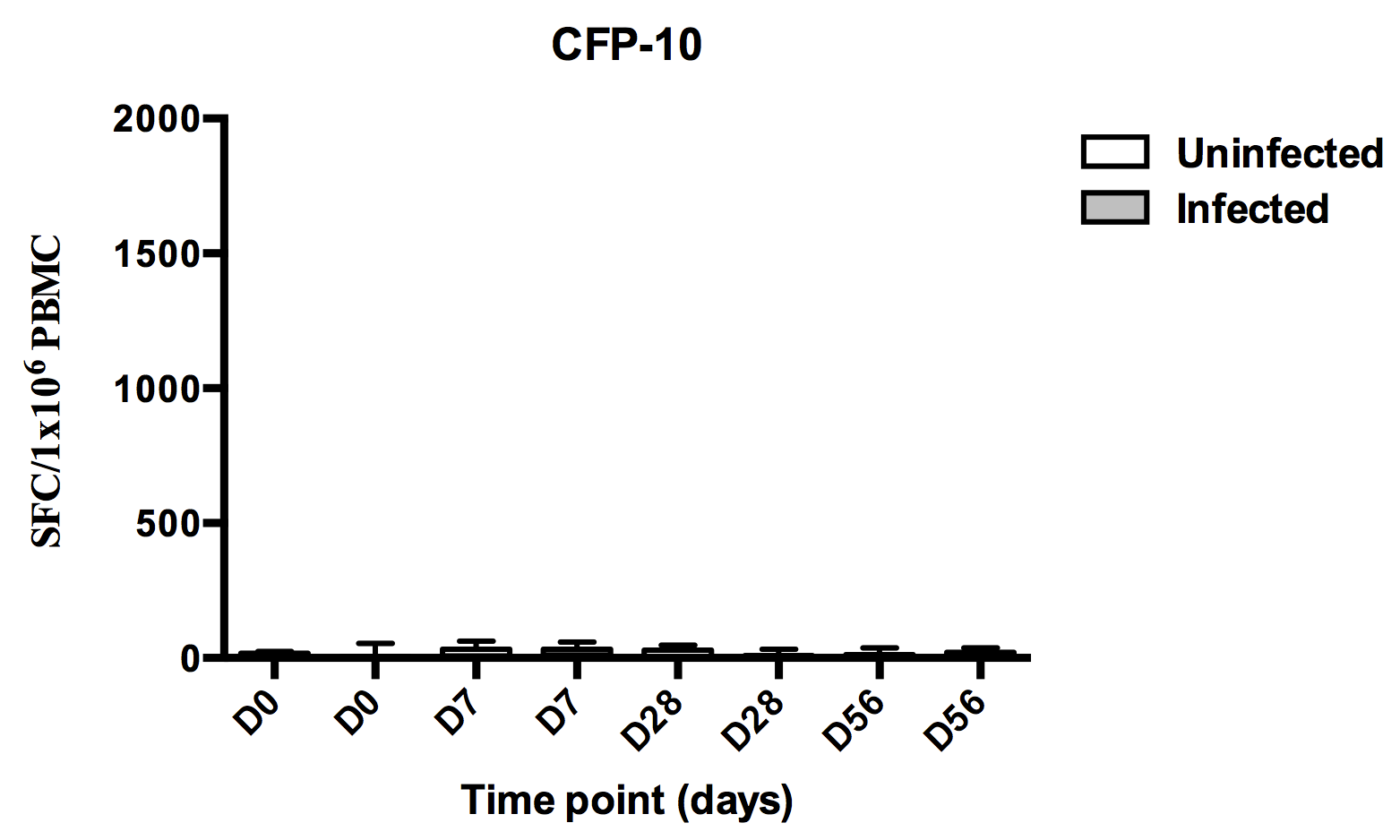

Supplement: S1 Fig — (DOCX) [file pntd.0005440.s002.docx]
