# Supplementary material for: The effect of current Schistosoma mansoni infection on the immunogenicity of a candidate TB vaccine, MVA85A, in BCG-vaccinated adolescents: An open-label trial
Source: PLoS Negl Trop Dis. 2017 May 4;11(5):e0005440. doi: 10.1371/journal.pntd.0005440 (PMC5417418; doi:10.1371/journal.pntd.0005440)
Supplement: S1 Table — (DOCX) [file pntd.0005440.s003.docx]

**Supplementary table 1. Regression Analysis of PPD response as measured by ELISPOT**

1. **PPD at day 7**

| **Factor** | **Level** | **Estimate (Bootstrap 95% C.I.)** | **P-value** |
| --- | --- | --- | --- |
| Constant |  | 129.5 (-2 751.8 ; 3010.9) | - |
| Age | Per one year increase | 4.7 ( -200.9 ; 210.2) | 0.96 |
| Sex | Male  Female | 0 (Ref)  29.9 (-516.5 ; 576.3) | 0.91 |
| School | Busabala  Officers’ children  Kigo-Lunya  Bulega  Bugiri | 0 (Ref)  -75.0 (-1 209.8 ; 1 059.8)  143.2 (-832.4 ; 1 118.8)  329.2 (-262.1 ; 920.4)  663.3 (-510.0 ; 1 836.6) | 0.25 |
| Hookworm Stool PCR | Negative  Positive | 0 (Ref)  -105.8 (-726.8 ; 515.1) | 0.74 |
| Helminth Group | No helminth  *Sm* only | 0 (Ref)  167.9 (-589.1 ; 925.0) | 0.66 |

Abbreviations: PPD, Purified Protein Derivative; C.I., confidence interval; PCR, polymerase chain reaction; *Sm, Schistosoma mansoni*; AUC, Area under the curve

1. **AUC for PPD (day 0 – day 28)**

| **Factor** | **Level** | **Estimate (Bootstrap 95% C.I.)** | **P-value** |
| --- | --- | --- | --- |
| Constant |  | -16 132 (-98 775 ;66 510) | - |
| Age | Per one year increase | 1 622 ( -4 221 ; 7 466 ) | 0.59 |
| Sex | Male  Female | 0 (Ref)  -74 (-11 740 ; 11 591) | 0.99 |
| School | Busabala  Officers’ children  Kigo-Lunya  Bulega  Bugiri | 0 (Ref)  1 048 (-19 895 ; 21 990)  7 379 (-11 560 ; 26 318)  7 964 (-7 152 ; 23 080)  9 941 (-11 638 ; 31 521) | 0.71 |
| Hookworm Stool PCR | Negative  Positive | 0 (Ref)  1 268 (-11 240 ; 13 776 ) | 0.84 |
| Helminth Group | No helminth  *Sm* only | 0 (Ref)  1 (-14 735 ; 14 736 ) | 0.99 |

Abbreviations: PPD, Purified Protein Derivative; C.I., confidence interval; PCR, polymerase chain reaction; *Sm, Schistosoma mansoni*; AUC, Area under the curve
