# Supplementary material for: The effect of current Schistosoma mansoni infection on the immunogenicity of a candidate TB vaccine, MVA85A, in BCG-vaccinated adolescents: An open-label trial
Source: PLoS Negl Trop Dis. 2017 May 4;11(5):e0005440. doi: 10.1371/journal.pntd.0005440 (PMC5417418; doi:10.1371/journal.pntd.0005440)
Supplement: S2 Table — (DOCX) [file pntd.0005440.s004.docx]

**Supplementary Table 2. Severity of graded adverse events**

|  |  |  | **Group 1** | **Group 2** | **Total** |
| --- | --- | --- | --- | --- | --- |
|  |  | **Severity** | **(No Helminths)**^a^ | **(SM only)** | **(All)** |
|  |  |  | **Number of** | **Number of** | **Number of events** |
|  |  |  | **events (%)** | **events (%)** | **(%)** |
|  | **All AEs** | Mild | 111(78) | 81(69) | 192 (74) |
|  |  | Moderate | 29(20) | 36(31) | 65 (25) |
|  |  | Severe | 2(1) | 0(0) | 2 (1) |
|  | **Total AEs** |  | 142 (55) | 117 (45) | 259 (100) |
|  | **Solicited AEs** |  |  |  |  |
|  | **Local AEs** | Mild | 39(85) | 34(79) | 73(82) |
|  |  | Moderate | 5(11) | 9(21) | 14(16) |
|  |  | Severe | 2(4) | 0(0) | 2(2) |
|  |  | Total | 46(52) | 43(48) | 89(100) |
|  | **Systemic AEs** | Mild | 62(82) | 39(68) | 101(76) |
|  |  | Moderate | 14(18) | 18(32) | 32(24) |
|  |  | Severe | 0(0) | 0(0) | 0(0) |
|  |  | Total | 76(57) | 57(43) | 133(100) |
|  | **Unsolicited AEs** |  |  |  |  |
|  |  | Mild | 10(50) | 8(47) | 18(49) |
|  |  | Moderate | 10(50) | 9(53) | 19(51) |
|  |  | Severe | 0(0) | 0(0) | 0(0) |
|  |  | Total | 20(54) | 17(46) | 37(100 |
|  |  |  |  |  |  |

Abbreviations: *Sm, Schistosoma mansoni;* AEs, Adverse events

a. Includes three participants subsequently excluded from the immunogenicity analysis due to misallocation
