# Supplementary material for: The effect of current Schistosoma mansoni infection on the immunogenicity of a candidate TB vaccine, MVA85A, in BCG-vaccinated adolescents: An open-label trial
Source: PLoS Negl Trop Dis. 2017 May 4;11(5):e0005440. doi: 10.1371/journal.pntd.0005440 (PMC5417418; doi:10.1371/journal.pntd.0005440)
Supplement: S3 Table — (DOCX) [file pntd.0005440.s005.docx]

**Supplementary table 3. Regression analysis of AUC comparing geometric means of cytokine responses to Ag85A between the uninfected and infected groups as measured by multiplex Luminex assay**

|  | **Unadjusted** | | |  | **Adjusted^1^** | | |  |
| --- | --- | --- | --- | --- | --- | --- | --- | --- |
| **Cytokine** | **Group 1**  **(No Helminths)**  **GM (95% C.I.)** | **Group 2**  **(Sm only)**  **GM (95% C.I.)** | **Mean**  **Fold Difference** |  | **Group 1**  **(No Helminths)**  **GM (95% C.I.)** | **Group 2**  **(Sm only)**  **GM (95% C.I.)** | **Mean**  **Fold**  **Difference** | **P value^2^** |
| IFN-γ | 9.35 [8.28,10.57] | 10.26 [8.90,11.82] | 1.10 |  | 9.58 [9.32,9.84] | 10.59 [10.09,11.12] | 1.11 | 2.890 |
| IL10 | 1.51 [0.61, 3.70] | 4.06 [2.55, 6.46] | 2.70 |  | 2.92 [2.13, 4.01] | 4.96 [4.51, 5.45] | 1.70 | 0.646 |
| TNFα | 7.60 [7.01, 8.24] | 8.55 [8.07, 9.05] | 1.12 |  | 7.69 [7.54, 7.83] | 8.59 [8.35, 8.84] | 1.12 | 0.289 |
| GMCSF | 8.07 [7.27, 8.95] | 8.83 [7.66,10.16] | 1.09 |  | 8.19 [7.83, 8.55] | 9.12 [8.82, 9.43] | 1.11 | 1.598 |
| IL12P40 | 4.80 [4.03, 5.70] | 3.72 [1.97,7.03] | 0.77 |  | 4.94 [4.39, 5.56] | 5.32 [4.64, 6.10] | 1.08 | 2.601 |
| IL13 | 6.15 [4.66, 8.13] | 8.05 [6.79, 9.54] | 1.31 |  | 6.71 [6.33, 7.11] | 8.45 [8.21, 8.69] | 1.26 | 7.004 |
| IL17A | 4.46 [2.25, 8.84] | 6.59 [5.62, 7.72] | 1.48 |  | 6.17 [6.01, 6.34] | 6.87 [6.70, 7.05] | 1.11 | 10.149 |
| IL1A | 3.07 [1.49, 6.31] | 3.18 [1.50, 6.76] | 1.04 |  | 4.57 [4.16, 5.03] | 5.32 [4.64, 6.10] | 1.16 | 0.969 |
| IL2 | 1.42 [0.63, 3.19] | 1.08 [0.47, 2.48] | 0.76 |  | 2.61 [2.16, 3.17] | 2.63 [2.38, 2.89] | 1.01 | 13.005 |
| IL5 | 4.22 [2.33, 7.63] | 4.68 [2.42, 9.05] | 1.11 |  | 5.59 [5.14, 6.08] | 6.85 [6.54, 7.18] | 1.23 | 2.210 |
| IP10 | 11.71 [11.31, 12.12] | 11.68 [10.99, 12.41] | 0.99 |  | 11.73 [11.63,11.83] | 11.76 [11.69, 11.83] | 1.00 | 15.657 |
| 15IP1ɑ | 7.16 [6.51, 7.89] | 8.26 [7.47, 9.14] | 1.15 |  | 7.26 [6.99, 7.54] | 8.42 [8.26, 8.59] | 1.16 | 0.595 |
| IL6 | 9.86 [9.18, 10.60] | 10.39 [9.86, 10.95] | 1.05 |  | 9.94 [9.66, 10.23] | 10.44 [10.19, 10.70] | 1.05 | 3.264 |
| IL12P70 | 0.87 [0.33, 2.25] | 1.59 [0.76, 3.31] | 1.84 |  | 2.00 [1.48, 2.72] | 2.67 [2.10, 3.40] | 1.33 | 0.935 |
| IL4 | 0.60 [0.24, 1.49] | 1.65 [0.78, 3.49] | 2.78 |  | 1.57 [1.22, 2.02] | 3.07 [2.68, 3.50] | 1.95 | 0.595 |
| MCP3 | 11.27 [10.62, 11.95] | 11.62 [11.18, 12.08] | 1.03 |  | 11.33 [11.09, 11.57] | 11.65 [11.44, 11.87] | 0.03 | 1.564 |
| MDC | 11.53 [10.98, 12.11] | 11.95 [11.61, 12.31] | 1.04 |  | 11.57 [11.34, 11.81] | 11.97 [11.84,12.10] | 1.03 | 3.094 |

Abbreviations: AUC, area under the curve; GM, geometric mean; Sm, *Schistosoma mansoni*; Ag85A, antigen 85A; C.I., confidence interval; IFN-γ, interferon gamma; IL, interleukin; TNFα, tumour necrosis factor; GMCSF, granulocyte-monocyte stimulating factor; IP, inducible protein; MIP1α, macrophage inflammatory protein-1-alpha; MCP3, monocyte chemoattractant protein-3 and MDC, macrophage-derived chemokine.

1. Age, gender and school
2. Adjusted for multiple testing using Bonferroni correction
