# Supplementary material for: The effect of current Schistosoma mansoni infection on the immunogenicity of a candidate TB vaccine, MVA85A, in BCG-vaccinated adolescents: An open-label trial
Source: PLoS Negl Trop Dis. 2017 May 4;11(5):e0005440. doi: 10.1371/journal.pntd.0005440 (PMC5417418; doi:10.1371/journal.pntd.0005440)
Supplement: S4 Table — (DOCX) [file pntd.0005440.s006.docx]

**Supplementary table 4. Regression analysis of AUC comparing geometric means of cytokine responses to PPD between the uninfected and infected groups as measured by multiplex Luminex assay**

|  | **Unadjusted** | | |  | **Adjusted^1^** | | |  |
| --- | --- | --- | --- | --- | --- | --- | --- | --- |
| **Cytokine** | **Group 1**  **(No Helminths)**  **GM (95% C.I.)** | **Group 2**  **(Sm only)**  **GM (95% C.I.)** | **Mean**  **Fold Difference** |  | **Group 1**  **(No Helminths)**  **GM (95% C.I.)** | **Group 2**  **(Sm only)**  **GM (95% C.I.)** | **Mean**  **Fold**  **Difference** | **P value^2^** |
| IFN-γ | 9.73 [8.92,10.62] | 11.17 [10.52,11.85] | 1.15 |  | 9.85 [9.62,10.09] | 11.23 [10.87,11.59] | 1.14 | 0.306 |
| IL10 | 2.98 [1.46, 6.08] | 5.71 [5.32, 6.14] | 1.92 |  | 4.09 [3.17, 5.27] | 5.76 [5.56, 5.97] | 1.41 | 1.190 |
| TNFα | 8.05 [7.59, 8.53] | 8.79 [8.45, 9.13] | 1.09 |  | 8.09 [8.04, 8.14] | 8.80 [8.59, 9.03] | 0.09 | 0.153 |
| GMCSF | 8.09 [7.56, 8.65] | 8.73 [8.13, 9.37] | 1.08 |  | 8.14 [7.85, 8.43] | 8.79 [8.48, 9.13] | 1.08 | 0.493 |
| IL12P40 | 5.62 [4.83, 6.54] | 6.51 [5.95, 7.12] | 1.16 |  | 5.79 [5.45, 6.14] | 6.59 [6.32, 6.88] | 1.14 | 1.462 |
| IL13 | 7.62 [6.92, 8.41] | 8.76 [8.15, 9.42] | 1.15 |  | 7.72 [7.39, 8.06] | 8.84 [8.53, 9.15] | 1.14 | 7.038 |
| IL17A | 7.25 [6.47, 8.12] | 7.72 [7.35, 8.10] | 1.06 |  | 7.39 [7.21, 7.56] | 7.75 [7.62, 7.89] | 1.05 | 0.476 |
| IL1A | 3.86 [2.22, 6.70] | 6.53 [5.78, 7.83] | 1.69 |  | 4.92 [4.47, 5.41] | 6.66 [6.23, 7.12] | 1.35 | **0.017** |
| IL2 | 1.33 [0.63, 2.84] | 1.27 [0.58, 2.79] | 0.96 |  | 2.32 [1.95, 2.77] | 2.69 [2.47, 2.94] | 1.16 | 6.647 |
| IL5 | 5.19 [2.95, 9.13] | 7.55 [6.91, 8.25] | 1.46 |  | 6.47 [6.01, 6.96] | 7.64 [7.28, 8.01] | 1.18 | 0.884 |
| IP10 | 11.95 [11.61, 12.30] | 12.09 [11.81, 12.38] | 1.01 |  | 11.97 [11.88, 12.06] | 12.10 [12.04, 12.17] | 1.01 | 8.925 |
| MIP1ɑ | 8.48 [7.80, 9.21] | 8.93 [8.43, 9.47] | 1.05 |  | 8.54 [8.10, 9.00] | 8.99 [8.75, 9.23] | 0.05 | 0.901 |
| IL6 | 11.69 [11.33, 12.07] | 11.82 [11.58, 12.08] | 1.01 |  | 11.71 [11.58, 11.85] | 11.83 [11.68, 11.99] | 1.01 | 3.077 |
| IL12P70 | 0.50 [0.19, 1.25] | 0.70 [0.30, 1.63] | 1.42 |  | 1.23 [0.68, 2.22] | 1.72 [1.31, 2.26] | 1.40 | 9.945 |
| IL4 | 0.64 [0.25, 1.61] | 1.28 [0.58, 2.82] | 2.02 |  | 1.78 [1.48, 2.14] | 2.70 [2.39, 3.06] | 1.52 | 10.421 |
| MCP3 | 12.04 [11.55, 12.54] | 12.34 [12.19, 12.48] | 1.02 |  | 12.07 [11.90, 12.24] | 12.34 [12.20, 12.47] | 1.02 | 0.425 |
| MDC | 11.63 [11.09, 12.19] | 12.09 [11.87, 12.30] | 1.04 |  | 11.66 [11.42, 11.90] | 12.09 [11.96, 12.22] | 1.04 | 0.884 |

Abbreviations: AUC, area under the curve; GM, geometric mean; Sm, *Schistosoma mansoni*; PPD, Purified Protein Derivative; C.I., confidence interval; IFN-γ, interferon gamma; IL, interleukin; TNFα, tumour necrosis factor; GMCSF, granulocyte-monocyte stimulating factor; IP, inducible protein; MIP1α, macrophage inflammatory protein-1-alpha; MCP3, monocyte chemoattractant protein-3 and MDC, macrophage-derived chemokine.

1. Age, gender and school
2. Adjusted for multiple testing using Bonferroni correction
